# Health professionals’ perceptions of how gender sensitive care is enacted across acute psychiatric inpatient units for women who are survivors of sexual violence

**DOI:** 10.1186/s12913-019-4812-8

**Published:** 2019-12-23

**Authors:** Carol O’Dwyer, Laura Tarzia, Sabin Fernbacher, Kelsey Hegarty

**Affiliations:** 1 Department of General Practice | Melbourne Medical School, Faculty of Medicine, Dentistry & Health Sciences, The University of Melbourne, Level 2, 780 Elizabeth Street, Melbourne, Victoria 3010 Australia; 2 The Royal Women’s Hospital, Centre for Family Violence Prevention, Melbourne, 3052 Victoria Australia; 3 Consultant, Melbourne, Australia

**Keywords:** Health professionals, Mental health care, Acute psychiatric inpatient setting, Trauma, Normalization process theory

## Abstract

**Background:**

Sexual violence is a global public health issue. It is a form of gender-based violence commonly experienced by women accessing mental health services. The biomedical model has been the dominant model of care in acute psychiatric units, however, there has been a global movement towards more gender-sensitive and trauma-informed models. To date, only a small amount of research has focused on evaluating these models of care and health professionals’ experiences of providing this care. The aim of this study is to gain an in-depth understanding of healthcare professionals’ perceptions of how Gender Sensitive Care (GSC) is enacted across acute psychiatric inpatient units for women who are survivors of sexual violence.

**Methods:**

This study used case study methodology and the Normalisation Process Theory (NPT) conceptual framework. NPT is a practical framework that can be used to evaluate the implementation of complex models of care in health settings. It included semi-structured interviews with 40 health professionals, document and policy reviews, and observations from four psychiatric inpatient units within a large Australian public mental health organisation. Data were examined using thematic and content analysis.

**Results:**

Themes were developed under the four NPT core constructs; 1) Understanding GSC in acute psychiatric units: “Without the corridors there’s not a lot we can do”, 2) Engagement and Commitment to GSC in acute psychiatric units: “There are a few of us who have that gender sensitive lens”, 3) Organising, relating and involvement in GSC: “It’s band aid stuff”, 4) Monitoring and Evaluation of GSC in acute psychiatric units: “We are not perfect, we have to receive that feedback**”.**

**Discussion:**

Many health professionals held a simplistic understanding of GSC and avoided the responsibility of implementing it. Additionally, the competing demands of the biomedical model and a lack of appraisal has resulted in an inconsistent enactment of GSC.

**Conclusions:**

Health professionals in this study enacted GSC to varying levels. Our findings suggest the need to address each NPT construct comprehensively to adequately implement GSC.

## Contributions to the literature

- Previous research has shown that the shift towards trauma-informed principles in mental health settings is encouraging, however, there is limited research to date evaluating the implementation and effectiveness of models of care in acute psychiatric inpatient units.
- This research provides qualitive insights using a case study methodology and NPT to further understand that challenges of implementing a complex model of care. NPT is a practical framework that can be used to evaluate the implementation of complex mental health models of care.
- These findings contribute to improving the provision of high quality, gender-sensitive care of female consumers during their admission to acute psychiatric inpatient units.

## Introduction

Sexual violence is a global public health problem and the health system has a key part to play in a multisectoral response [1]. Primarily perpetrated by men against women, sexual violence has strong associations with poor mental health [2], including serious mental illness [3, 4]**.** It is estimated that one in three women accessing inpatient or outpatient mental health services have previously experienced domestic violence, including sexual violence [5]. Despite this, historical sexual violence is generally not identified by mental health services [3]. Furthermore, international research suggests that mental health services have tended to overlook the privacy and safety needs of female patients in psychiatric settings [6]. Women consumers in inpatient units frequently report experiencing harassment, intimidation, abuse, feeling threatened or unsafe [7]. Currently in Australia, most psychiatric units continue to have mixed gender wards [8, 9]. Staff report a lack of confidence and skills to respond appropriately to disclosures and therefore frequently do not ask about sexual violence [10, 11], whether historical or experienced on the ward. This avoidance of sexual violence is further compounded by the dominant biomedical model that informs mental health services [9]. The biomedical model views trauma symptoms as pathological rather than adaptive [12], and focuses on diagnosis, treatment, and prescription of medication [13, 14].

Encouragingly, there is a global shift across mental health settings from the biomedical model [15] to more trauma and gender inclusive models of care to support consumers with various presentations and co-morbidities [16–21]. Trauma-informed care (TIC) recognizes the responsibility of mental health services to be responsive to the pervasiveness, bidirectionality and impact of trauma experienced by people with mental health disorder at a systemic level [22]. GSC is a multi-elemental model of care, within TIC, that specifically considers gender and individual factors in the context of service provision [16]. However, implementing these models of care in acute psychiatric inpatient units is complex, resource-intensive and requires a whole of systems response [1]. Despite training and education, health professionals continue to struggle to translate the values and principles into their day to day practice [23]. Previous work in this area suggests that in order for a model of care to be effective, it must be integrated into all levels of policy and practice to influence how services and staff care for female consumers [24] and be in line with the values of staff implementing it [25, 26]. Moreover, it needs to acknowledge and address the tensions that exist between the biomedical model, including compulsory treatment, control, risk and safety with the principles of gender sensitive and trauma-informed care, such as collaboration and choice [27]. Yet, existing research would suggest that this has not yet occurred within the acute psychiatric inpatient setting in Australia.

There is a paucity of literature that evaluates the implementation and effectiveness of trauma-informed models of care at an organisational or systems level [28]. There is also an underdeveloped systems approach to implementing GSC [16], specifically in clinical psychiatric settings [29]. Most research on trauma-informed care to date has focused on reducing restraint and seclusion and utilized quantitative approaches [30] . It has also focused on examining the challenges to implementing trauma-informed care in acute mental health settings and mapping these findings against the principles of trauma-informed care [30]. The aim of this study is to gain an in-depth understanding of health professionals’ perceptions of how GSC is enacted across psychiatric inpatient units for women who are survivors of sexual violence. We draw on rich qualitative data, document and policy reviews and observations, and use Normalisation Process Theory (NPT) [31] to assist with interpreting our findings. NPT is a social implementation theory [31] that recognises the challenges of implementing complex interventions in healthcare systems with multiple professions and interactions [32]. NPT provides a conceptual framework for understanding how new and complex interventions become *normalised* into clinical practice [33] through implementation, embedding and integration [31]. NPT can be used to design interventions or organise and evaluate interventions as part of the implementation process [32]. The knowledge generated by this study is integral to understanding and improving the provision of high quality, gender-sensitive care of female consumers during their admission to acute psychiatric inpatient units.

## Method

### Context

The Victorian Government began to focus on GSC as a model of care following reports by women in psychiatric inpatient units of feeling unsafe and experiencing sexual violence [10]. This resulted in the publication of ‘The gender sensitivity and safety in adult acute inpatient units’ project report and the ‘Promoting sexual safety, responding to sexual activity, and managing allegations of sexual assault in adult acute inpatient units’ guideline [34]. Furthermore, the ‘Service Guideline on Gender Sensitivity and Safety: Literature Review’ [35] and ‘Service guideline on gender sensitivity and safety; Promoting a holistic approach to wellbeing’ were published in 2011 [36]. The latter was implemented by the mental health organisation included in this study following its publication. This guideline specifically provides practical directions on gender-sensitive care, trauma-informed care, bed-based services and responding to incidents [36]. It also provides directions for supporting those who have experienced various forms of violence and diverse populations and communities, including women with mental illness and trauma histories [36]. The gender sensitive model of care operates at an organisational and systems level, including the environment, management and leadership, direct contact staff, practitioner support, referral pathways, information sharing, protocols and policies, and community linkages [36].

### Study design

This study used a qualitative case study design as its methodology of inquiry. Case study methodology involves the investigation of a phenomenon within its real-life context [37]. A collective instrumental design with cross-case analysis [38] was chosen as the most suitable to illustrate the similarities and differences of this case study. This case study specifically explores health professionals’ perceptions of providing GSC for women who are survivors of sexual violence within four different acute psychiatric inpatient units within one mental health organization in Victoria, Australia. It involved semi-structured interviews, document and policy analysis, and observations over a 12-month period.

### Recruitment

Health professionals were recruited from a variety of different professions, levels of seniority, and genders using a purposive sampling strategy. Approval was sought initially from managers, the lead researcher then attended staff meetings to promote the study and distribute Expression of Interest forms. Interview times and dates were arranged at a mutually convenient with staff who expressed interest. For de-identification purposes all participants have been provided with a pseudonym.

### Participants

Forty mental health professionals participated, including 20 psychiatric/nursing staff, 10 allied health professionals (psychologists, social workers and occupational therapists), seven medical staff (psychiatric registrars, medical officers and consultant psychiatrists) and three consumer/peer support staff. Staff in psychiatric inpatient units undergo specialization in mental health/illness prior to working in these settings. Twenty-seven participants identified as female and thirteen as male. Thirty-one staff worked full-time and nine part-time. Participants had varying levels of experience, ranging from three months to 29 years. Their age varied from 21 to 64 years with a mean of 42.5 years. Staff predominantly identified as being of Australian nationality (77.5%). Staff had access to training on gender sensitivity and safety as part of the implementation process of the gender sensitivity and safety guideline [36]. Participants names have been changed to pseudonyms’.

### Data collection

Forty semi-structured interviews by phone [2] or face-to-face [38] were conducted between March and December 2016. The female lead researcher (COD), who is a registered psychologist and PhD Candidate, conducted all interviews individually in a private office space across the four psychiatric inpatient units. Confidentiality, anonymity, the research aims and rationale were reiterated prior to commencing the interview to reduce social desirability bias [39]. The semi-structured interview involved pre-determined open-ended questions but also allowed for discussion with the participants. Interviews ranged from 12 to 90 min (average of 40 min), covering questions on the type of care provided to women with a history of sexual violence, how care is implemented, and how it is supported (see Additional file 1 for further information). Questions were pilot tested with allied health professionals not employed by the Health Service. Field notes were made before, during and after the interviews. Observations were also conducted at the four psychiatric inpatient units over the 12-month period. All interviews were audio recorded and fully transcribed verbatim by a professional transcription service. Participants names have been changed to pseudonyms by the researcher. These are used, along with professional background, to support quotations and give context. Feedback on transcripts was offered to all participants, two participants provided comments and corrections on their transcripts. Documents were also collected from the mental health organization and specifically from all four acute psychiatric units.

### Data analysis

The documents and transcripts were coded by the lead researcher using NVivo 11 (QSR International, 2015) software to manage the data. The lead researcher (COD) used content analysis to analyse the documents [40]. Analysis focused on understanding health professionals’ experiences of how gender-sensitive care is enacted across acute psychiatric inpatient units for women survivors of sexual violence. It also focused on understanding the experiences and perceptions of providing this care [41]. The lead author engaged in regular reflective practice and supervision with co-authors throughout the analysis and coding process. The lead researcher used a inductive method to code all the interview transcripts and conduct a thematic analysis [42]. Relevant statements were coded with ample context to avoid data fragmentation and decontextualisation [43]. The authors (KH, LT, SF) worked collaboratively to discuss the emerging themes, resolve any differences and to ensure consensus on the development of these themes [44]. These themes were mapped to the corresponding NPT core constructs. NPT is operationalised through four main constructs; Coherence-how participants make sense of the intervention, Cognitive Participation- commitment and engagement of the intervention by participants, Collective Action- organising and enacting the intervention and Reflexive Monitoring- how participants appraise the intervention after it is in use [31].

### Ethical considerations

The study was approved by both the Human Research Ethics Committee (HREC) at Melbourne Health and the HREC at the University of Melbourne. Participation was confidential, and managers were not informed about who participated. Informed consent was obtained via a Plain Language Statement. Participants were asked to sign and return a consent form prior to commencing an interview. No incentive was offered.

## Results

The findings from the health professionals’ interviews, documents and policy reviews, and observations are outlined below within the four NPT core constructs, relating them to the main findings in each construct. No major differences were found across the four different psychiatric inpatient units following data analysis, thus the similarities between the four units is discussed below.

### Understanding GSC in acute psychiatric units: “Without the corridors there’s not a lot we can do”

This construct refers to an individual and communal understanding of the intervention or process required and how it differs to previous work. For an intervention such as GSC to be normalised, all stakeholders need to understand and value the model of care and to demonstrate some investment in the *meaning* of it. Most of the health professionals in this study make sense of GSC predominately through the physical characteristics of the units and the biopsychosocial treatment modalities provided.

Health professionals across all four inpatient units appeared to have a similar understanding about what GSC is, with consistent use of documents across all four units. Many of the staff described the physical aspects of the units such as the segregation of genders through women only corridors and women only groups, and the provision of swipe wrist bands to access the women only corridors. As Chris (nurse) described “*The female corridor can be locked off, the consumer gets a swipe [band] to swipe in and out of that locked area. I think if anything we’ve made advances in that area, being a bit more protective of our vulnerable females. We’ve also got women’s only lounges for the girls if they don’t feel comfortable sitting in the lounge area with multiple people. It’s definitely the biggest difference in recent years”.* Several staff also identified the ability to monitor and observe women who were deemed more vulnerable as part of GSC. They described doing this by placing them in closer proximity to the nurses’ station if a bed wasn’t available in the women’s corridor. Alternatively, women were moved to another area of the ward following an incident or allegation, however, this was not always possible as described by Jason (Allied Health) “*The perpetrator and victim had to stay in the locked ward together, so we had to get extra nursing staff to observe the perpetrator. Once the victim became better, she was transferred to her own space”.*

Treatment was described as providing individualised treatment plans, including sensitive physical examinations if needed. It includes attempts to manage risk through frequent risk assessments and minimise retraumatisation through a reduction in restrictive intervention. Bernadette (Nurse) reported *“throughout your shift you do a constant risk assessment*”, furthermore Lucy (Nurse) described the care as *“coordinating restraints, injections and bedroom allocations, avoiding placing a female consumer beside a male who is disorganised”.* Treatment modalities reportedly ranged from *“medication that we use through to sensory modulation. The occupational therapy team has the best expertise in that. Psychology if it’s appropriate. We will sometime make safety plans. In terms of day to day care and making sure the staff are aware of any anxiety that they may have, and they are attentive to that”* (Jack, Medical). Some staff also reported that this treatment planning extended to internal referrals, and external referrals as part of discharge planning. Internal linkages between different disciplines (OT, psychologist, social worker, peer support staff) and external referrals (Police, sexual assault services, community services) were made to ensure appropriate support regarding sexual assault or safety issues. Gillian (Nurse) described the care provided to a women who was admitted who had recently been sexually assaulted *“she was admitted to our intensive care area, started on medication for her mental state, became better and was supported to get in touch with (sexual assault service) contacting the police and the (sexual assault police unit) interviewed her on the ward, she got better and went ahead with the charges”.*

### Engagement and commitment to GSC in acute psychiatric units: “There are a few of us who have that gender sensitive lens”

This construct states that normalisation of a new practice or model occurs if all stakeholders engage with the model and use it and that there is a demonstration of commitment to GSC required to implement the intervention. Participation in the implementation of GSC was dependent on health professionals’ understanding of how they make sense of GSC as described above and their commitment to it. Female staff and middle management recognise the importance of GSC as part of their role. However, many staff described it as “others” role.

Many of the health professionals described female staff as key participants in providing GSC *“We do try to accommodate the patient by trying to get female clinicians or doctors”* (Frank, Medical). These also included staff with specialist knowledge in GSC, such as the “creating safety” nurse on each ward, staff who were part of the gender sensitivity and safety committee at a unit or organisational level, allied health staff and peer support staff. *“Allied Health’s really good. Their understanding of issues and their awareness is greater. Our creating safety nurse on the unit’s an enabler. I recently lobbied to have a women’s group reinvigorated for consumers around their safety on the units and having consumer peer workers involved and that was really warmly received by the coordinator, the peer workers themselves and the nurse unit manager”* (Kaye, Allied Health). Middle management, such as clinical managers, nurse unit managers, shift leaders, nurse in-charge were also identified by staff as being key participants in implementing GSC on the units. It was described as the responsibility of specialist staff and middle management to collaborate and consult with external services, such as sexual assault services. This collective response to an incident or allegation was described by Bernadette (Nurse) *“The social worker would be involved, the treating team on both clients, and the team meaning there’s a psychiatrist and a doctor, whether it’s a hospital medical officer, whether it’s a registrar, or an intern. The nurse in charge gets involved somewhat, the contact nurse definitely gets involved”.* However, only one unit reported identifying Executive Management as driving implementation and having strong linkages with external services. This deficit was recognised by James (Nurse) *“It flows down from management and senior clinicians, whether it’s doctors, nurses… it trickles down into culture to support and take these incidences seriously”.*

Key staff, described above, recognised GSC as part of their role, however, most staff dismissed it as the role of others. Male staff avoided responsibility for participating in GSC due to it being “secret women’s business”. Many of these health professionals from various professional backgrounds blamed their inability to participate in GSC on others and external factors rather than reflecting on their own personal reasons or deficits. Examples of this include not having a psychologist on staff, a lack of staff with specialist knowledge or having to follow directions by leadership or management as getting in the way to them being able to implement GSC. Some staff also blamed process issues, such as lack of multidisciplinary involvement and collaboration during handover, ward rounds etc. *“The doctors are working so intensely with someone I think they forget the scope of what other disciplines can offer”* (Alison, Allied Health). Many staff recognised a major reason for this othering was due to a high percentage of new staff, students and interns who lack the training and skills to implement GSC.

### Organising, relating and involvement in GSC: “It’s band-aid stuff”

This construct refers to doing the work needed to enact a set of practices. This includes how new roles are organised, how people relate and are involved, and the knowledge and skills needed to complete the work. Normalisation of a model involving GSC occurs if all stakeholders work to operationalise it and demonstrate investment in effort. Staff reported many challenges to operationalising GSC, such as the physical barriers, consumer presentations, bed pressure, quick discharge of consumer, prioritising administrative tasks with time spent with consumers etc. These challenges highlight the dominance of the biomedical model in mental health which thereby impacts who is involved in the work and how the work gets done. Staff expressed the importance of collaboration, drawing on specialist knowledge direction and training as highly important to encouraging staff to enact and maintain involvement in GSC. These issues were further reinforced by an organisational emphasis on reactivity to incidents, a lack of consistency and commitment to enacting GSC.

Many staff expressed physical challenges to enacting GSC that were in contrast to the physical aspects they described as how GSC is enacted e.g. women’s only corridor. These included physical barriers of the wards, such as mixed gender wards, small environments with limited space to move around, shared rooms and bathrooms, and the layout with the nurses’ station overlooking the unit and therefore not engaging with consumers. Kelly (Health professional) comments on the overall issue *“The environment is crucial to improving mental health, so I think there really needs to be a lot of changes around the way mental health units are designed. And because I used to work at [another hospital] there was a lot of work done on the design of the hospital, and just integrating the natural environment into the hospital environment. I think there is a lot of improvement needed in that area”*. Nursing staff frequently identified an uncertainty of prioritising daily unit tasks and duties, such as administration, with apportioning time to building relationships with consumers, which is key to GSC. This was reflected by Elise (Allied Health) *“The nurses try really hard to be on top of it which is impossible because they are so busy and the demands of day to day tasks of observing and medication and nursing care and a zillion other thing”.* This time pressure and burden of tasks resulted in responding to consumers at a reactive level as per *‘squeaky wheel’* rather than providing individualised proactive care to all consumers.

Health professionals reported an increase in acuity and aggressive behaviours from consumers due to substance use, namely ICE. This resulted in a retraumatising and countertherapeutic environment. Keith (Nurse) provided an example *“At 2am in the morning and people are screaming, carrying on. Fires are being lit in the ward, people are being assaulted. The police are here, the fire brigade are here. I know the management are quite supportive, but I think it’s a different thing. Being supportive against being there in the moment and experiencing those sorts of things”*. Another pressure reported by most staff was to stabilise the consumers mental health symptoms and to discharge. Camilla (Nurse) stated *“It’s very much a band aid here, you just stabilise them and send them out and hope for the best. I don’t think it would be that great, especially in ED, I did a couple of emergency shifts over the weekend and apparently if anyone comes in with issues related to sexual violence issues, no-one in ED here deals with it, they have to go to the [another hospital]”.* A lack of collaboration with other hospitals, community or external services also impedes enactment of GSC or long term follow up. Furthermore, staff reported pressure to prioritise bed availability and filling beds over safety of women or ensuring women were allocated to women only corridor. Due to the limited number of women only corridor beds, staff were required to assess women’s vulnerability using a checklist and allocate accordingly. Observations at meetings and on the units supported this pressure to prioritise discharge of consumers and allocate beds to admissions. Lauren (Nurse) described “*I think there’s some challenges, we’ve got the guidelines, the flowchart that prioritises, which women will go into the female corridor, sometimes we will just have women say, “No, I don’t want to be moved, I want to stay here.“ In the end we have to respect someone’s choice. There’s always challenges I think, just in terms of staff awareness, and being more attuned to those risks*”. This struggle was evidenced in documents that aided in decision making and prioritising of women to women’s only corridors based on assessment needs.

Staff recognised the importance of a supportive team environment and collegiate support to overcome some of these daily challenges and concerns. This was evident at various levels and across all professional backgrounds. This was maintained through good communication on daily issues, regular unit meetings and good linkages with specialist services and staff, regular training, information sharing and clear resources. This maintained individuals’ motivation and engagement in participating and practicing GSC. For junior staff having regular access to supervisors was important. Nursing and allied health staff reported being able to approach colleagues with specialist knowledge and those on the gender sensitivity and safety committee for advice and feedback. Allied Health staff appreciated being collaborated with at a multidisciplinary level. Kate (Allied Health) commented *“I have good supervisors. They provide really good support. We have a good multidisciplinary team. We’re quite good at bouncing ideas off each other, just coming up with ways in which we can further support the person. If we’re hitting dead ends, I know a lot of the nurses are the main contacts for people. They get quite a lot of information”.* Medical staff and those at middle management level reported supportive leadership and being able to seek out clinical governance advice was extremely beneficial. *“Just get some advice from my seniors to see if there’s something more to be done”* Frank (Medical) commented. Overall most of the health professionals in this study reported working collaboratively was highly motivating and provided job satisfaction.

Many health professionals recognised that having clear procedures, protocols, guidelines and evidence-based practice, regular training as useful in enacting GSC. This was supported by the consistent use of policies and procedures promoting gender sensitive practice and sexual safety across the mental health organisation. Furthermore, clear reporting avenues and documentation enabled staff to share information from one shift to the next. However, this was also seen as a downfall if staff did not clearly document then it was often overlooked as described by Brooke (Nurse). *“If there’s an incident on the ward where sexual activity has taken place, we have protocols in place. We have a clear outline of what needs to happen and what has to happen. It just depends on which nurse is on, as to whether it happens or not. Management will follow it up. All incidences. Make sure everything gets [reported on the internal database]. Make sure that the proper channels are gone through”.* The gender sensitivity and safety committee provided a regular opportunity for key drivers from each unit to collaborate to work together on GSC topics *“Having champions that will hold that information and that’s where the Gender Sensitivity Committee’s become really important. We’ve always got that lens in our work, but how do we have other people have that lens in their work as well? That’s the challenge”* commented Maryann (Allied Health). Developing all health professionals knowledge and skills in GSC through forums, training, education and staff meetings where gender sensitive topics were raised was highly valued by staff. This was particularly important for new graduates and junior staff who lacked the skills and experience to confidently respond to gender sensitive issues.

It was evident across all four units that engagement with GSC and therefore implementation varied depending on key drivers. Some documents across the four units were consistent however this was not always the case, which contributed to the varied staff involvement. At an organisational level, staff reported that senior staff were not involved in the practical implementation of gender sensitive care model. *“That’s one of the things I’ve thought the [gender sensitivity and safety] committee fell down a bit because these came out of that committee but they weren’t really endorsed by the Executive, the whole organisation, I think we need the Exec to push this stuff”* (Kaye, Allied Health). Furthermore, individual units and key drivers were required to build relationships with other services (Police and sexual assault services) to effectively work together. This was resource intensive and again dependent on the priorities of each unit.

### Monitoring and evaluation of GSC in acute psychiatric units: “We are not perfect; we have to receive that feedback”

This construct is the individual or group assessment of the way new practice affects participants and other stakeholders. Normalisation occurs if continual monitoring and evaluation of GSC occurs. It refers to an investment in *comprehension* (May et al., 2011). At an individual unit and organisational level, evaluation and appraisal of GSC was reactive and in response to incidents that occurred. This reactive appraisal highlights the prioritising of safety and risk management that inadvertently reduces the priority of the principles of choice and control, women’s or gender specific needs.

Monitoring and evaluating GSC was dependent on the priorities and the key drivers of each unit. This was predominately at a reactive level, with middle management health professionals reporting implementing recommendations and changes based on review of incident reports, presenting and receiving feedback at a forum level. The review of a key sexual safety policy was delayed due to the involvement of an external organisation. Furthermore, staff identified opportunities for reflective practice to be minimal or reactionary to an incident i.e. diffusion/ debriefs post incidents of sexual violence. Staff reportedly appreciated and benefitted from these opportunities when offered. Jason (Allied health) described *“We do have regular diffusion sessions and reflective practice sessions on this ward. The diffusion is after an incident has occurred but reflective is where we are encouraged to open up about our feelings. There are so many things that happen on a ward on a regular basis that we need the opportunity to get it off our chests”*.

Appraisal of the effectiveness and usefulness of GSC was reported to be minimal. The feedback sought was often reactive with each unit reportedly contacting consumers post discharge however limited feedback was sought regarding safety, choice, control etc. Charlotte (Management) described how this was carried out on her unit “*taking the feedback and/ or complaint and talking to the consumers and staff, figuring out what went wrong and how we can improve that process”.* One unit did report to have held a focus group of female consumers some years ago to seek input into the development of a document. However, this appeared to be the only proactive appraisal reported.

At an organisational level, data was collected via a risk reporting database that categorised incidents of sexual violence. Those reported to be of a serious level were reviewed by senior management. These incidents reviews lead to discussion at a middle management, an individual staff or staff meeting level depending on what was needed. This lengthy process was describe by Steven (Management) *“Once an incident has been reported at level two, we review it internally and look at the rating of the event and the incident and determine the level of review that’s required, for a more serious incident we would do an in-depth clinical review, for a medium rating it might be a team based review and then for a more straight forward one it might be just to ask the manager to review internally, the review findings and recommendations have to be reported to the board committee, which oversees safety”.* Attempts to refine procedures and modify practices again reportedly occurred on a reactive basis, depending on outcomes of incident reviews and via external sources.

## Discussion

The aim of this study was to understand health professionals’ perceptions of how GSC is enacted in acute psychiatric inpatient units. This was undertaken using a case study methodology [37] and the NPT framework [31] to guide analysis. Overall, we found that staff understand GSC in simplistic terms. A significant challenge to implementing GSC is health professionals’ lack of understanding of the complexity of this model of care. GSC is a multi-element model of care that considers gender, and individual factors in the context of service provision [16]. It is based on the principles of safety, knowledgeable staff, respect, acceptance and the avoidance of re-traumatization [16]. These challenges are perpetuated by the biomedical model’s focus on symptoms and the constant pressures to manage compulsory treatment, control, risk and safety in psychiatric inpatient units [13]. The development of women’s only corridors was initially used to highlight the need for safety [45], but has now become the main intervention that accompanies the biopsychosocial interventions routinely utilised in acute psychiatric units.

This study found that key drivers and working as a team were essential in participating and enacting GSC in all four units. However, a significant number of health professionals’, predominantly male staff, justified their discretionary involvement in GSC. As a result, the role of enacting GSC was designated to key health professionals with specialist knowledge and/or female staff to implement a systems level model of care. This diffusion of responsibility has been linked to role, gender and health professionals’ years of experience [41]. Furthermore, this perspective results in a lack of systems or organisational approach to implementation and thereby individual units implementing GSC inconsistently depending on the motivation of the key drivers. The appointment of ‘champions’ would be a proactive way of supporting all staff within the organisation to implement GSC and improve cultural change to address violence against women [46]. Furthermore, health professionals across the organisation need a shared understanding of roles and language, strong relationships between teams, regular training with feedback mechanisms and mandatory or motivated training attendance [46]. Importantly, this training needs to be targeted at the appropriate level depending on individual health professionals’ knowledge, attitudes and skills of GSC [41]. Another challenge for staff was enacting GSC whilst balancing the competing demands of an acute psychiatric inpatient unit. Successful implementation of healthcare-based interventions needs to be tackled at an organisational level. Commitment needs to come from executive leadership and involvement from the bottom up to promote GSC, trauma-informed principles and gender-equitable attitudes [1]. The organisation also needs to establish clear referral pathways between health care services and special sexual violence services [1].

Monitoring, evaluation and appraisal was shown to be reactive at an individual unit and organisational level in this study. This is a major deficit to adequately implementing GSC and within mental health organisations more broadly [47]. Monitoring and appraisal is essential to strengthen a health systems response to address violence against women [1]. It aids progress at an organisational level by feeding back referral data, monitoring progress of consumers, quality of care provided, funding requirements but also at a systems level to provide feedback on whether the model of care is effective or not [1]. Services must collect information to inform policies, monitor services and improve the care they are providing. In order for GSC to be normalised, all stakeholders need to have a comprehensive understanding, engage with the model of care and use it, work to operationalise it and demonstrate investment in effort, and continual monitoring and evaluation [31]. This study has shown that many of these important features are lacking and therefore will continue to be a barrier to providing quality GSC.

## Limitations

Although participants were recruited from four different psychiatric inpatient units, these were all within the one mental health organisation. This sample is likely to have been informed by health professionals who had a greater knowledge of gender-sensitive issues than other mental health organisations. Researcher bias was minimised through co-development of the coding framework and collaborative review of themes between four members of the research team (each drawing on different disciplinary backgrounds). However, it is still possible that the experiences of the research team influenced our interpretation of data.

## Implications

Further research is needed to identify and evaluate effective models of care to target the intersectionality of mental health and sexual violence within the healthcare sector. Additionally, there is a need for a functional health system response, with a multi-element response that addresses violence against women [1] and incorporates comprehensive monitoring and evaluation of the care provided [48]. This is an ongoing challenge in Australia and internationally due to frequently changing political landscapes and priorities [1].

## Conclusion

These findings highlight the challenges of implementing a new model of care within the biomedical model of mental health organisations in Australia. To date, there has been limited qualitative research into health professionals’ perceptions of care provided in this setting or evaluation of the model of care being implemented. This is problematic as it has expected health professionals to understand, participate, enact and appraise a new model of care that is incongruent to the overarching biomedical model that governs their practice. Most research investigating mental health and trauma has centred around the reduction of restrictive interventions and seclusion, practical strategies of implementing trauma informed care, changes to leadership-styles, modification to the historical authoritarian nurse-consumer relationships and changes to policies [30]. As we can see a multi-element healthcare systems response is needed to adequately address violence against women with associated mental health difficulties and monitor its effectiveness [1]. Further research could include understanding consumers’ perceptions of attending acute mental health and the models of care they expect.

## Supplementary information

**Additional file 1:** The GSC Project Semi-Structured Interview Guide.

## Data Availability

The datasets used and/or analysed during the current study are available from the corresponding author on reasonable request.

## Abbreviations

GSC: Gender Sensitive Care
NPT: Normalisation Process Theory
TIC: Trauma Informed Care
