# Supplementary material for: Health professionals’ perceptions of how gender sensitive care is enacted across acute psychiatric inpatient units for women who are survivors of sexual violence
Source: BMC Health Serv Res. 2019 Dec 23;19:990. doi: 10.1186/s12913-019-4812-8 (PMC6929426; doi:10.1186/s12913-019-4812-8)
Supplement: Supplementary file 1 — Additional file 1: The GSC Project Semi-Structured Interview Guide. [file 12913_2019_4812_MOESM1_ESM.docx]

**The GSC Project**

**Semi-Structured Interview Guide**

**Introduction**

- Introduce interviewer and project;
- Ask for responses to consent form questions (e.g. regarding confidentiality, attribution, and recording);
- Retain signed copy of Consent Form.

**Semi-Structured Interview Questions**

1. **Can you tell me about your profession and role within this AMHS/unit**?
2. **Can you tell me about the care that you (is) provide(d) for women in the inpatient unit who have previously experienced unwanted sexual contact?**
3. **How supported do you feel to deliver this care?**
4. **Can you tell me your understanding of GSC?**
5. **Could you describe barriers, challenges and enablers of (in supporting the) implementing GSC?**
6. **How do you think the female patients experience/ feel about the care they receive?**
7. **Is there anything else you would like to add?**

Thank you again for participating.

Should you become distressed or affected by our interview please remember that there is a list of numbers you can contact for professional advice on the resource card.
